# Cost Savings and Reduced Health Care Utilization Associated with Participation in a Digital Diabetes Prevention Program in an Adult Workforce Population

**DOI:** 10.36469/jheor.2020.14529

**Published:** 2020-08-18

**Authors:** Cynthia Castro Sweet, Carolyn Bradner Jasik, Amy Diebold, Ashley DuPuis, Bryan Jendretzke

**Affiliations:** 1 Medical/Clinical, Omada Health, Inc., San Francisco, CA; 2 IBM Watson Health, Ann Arbor, MI; 3 The Dow Chemical Company, Midland, MI

**Keywords:** diabetes prevention, health care utilization, digital health, cost savings

## Abstract

**Background:**

Though in-person delivery of the Diabetes Prevention Program (DPP) has demonstrated medical cost savings, the economic impact of digital programs is not as well understood.

**Objective:**

This study examines the impact of a digital DPP program on reducing all-cause health care costs and utilization among 2027 adult participants at 12 months.

**Methods:**

A longitudinal, observational analysis of health care claims data was conducted on a workforce population who participated in a digital diabetes prevention program. Differences in utilization and costs from the year prior to program delivery through 1 year after enrollment were calculated using medical claims data for digital DPP participants compared to a propensity matched cohort in a differences-in-differences model.

**Results:**

At 1 year, the digital DPP population had a reduction in all-cause health care spend of US$1169 per participant relative to the comparison group (*P* = 0.01), with US$699 of that savings coming from reduced inpatient spend (*P* = 0.001). Cost savings were driven by fewer hospital admissions and shorter length of stay (*P* < 0.001). No other significant results in cost differences were detected. There was a trend toward savings extending into the second year, but the savings did not reach statistical significance.

**Conclusions:**

These results demonstrated significant short-term health care cost savings at 1 year associated with digital DPP program delivery.

## BACKGROUND

Type 2 diabetes (T2D) is a pervasive and costly condition in the United States, with over 30 million Americans diagnosed with T2D, and an additional 84 million at risk for developing the chronic condition.^1^ The economic cost of diabetes is estimated at US$327 billion dollars (in 2017 US dollars) in combined direct medical costs and indirect costs.^2^ The economic burden of the condition on employment and employers is well known: diabetes results in lost labor productivity due to absenteeism and presenteeism, and loss/removal of laborers from the workforce.^3^,^4^

More employers are implementing efforts to prevent diabetes to improve the health status of the workforce and curtail future health care spending. The Diabetes Prevention Program (DPP) is an evidence-based lifestyle management program proven to reduce the risk of diabetes among people with prediabetes through improved eating patterns, physical activity, and weight loss; at three years, the DPP has been shown to reduce diabetes by 58%.^5^

The DPP has widespread dissemination across various settings, with replications of success in reducing population risk for T2D and cardiovascular disease.^6^,^7^ Specific worksite-based DPP adaptations have been deployed with mostly successful results.^8^–^11^ Despite demonstrated impact on diabetes risk and cost savings, access to the DPP program has been limited and the preventive service is not reaching its potential.^12^ The Centers for Disease Control (CDC) and the American Medical Association (AMA) recommend the DPP for all adults who meet the criteria for prediabetes, but adoption of the program has been limited by program availability, reimbursement, and provider knowledge.^13^,^14^

Digital delivery of the DPP can increase access to the program because of the ability to reach users at any time or in any location. Eighty one percent of US adults now own a smartphone.^15^ This makes it possible for virtually any patient to be reached. Digitally adapted versions of the DPP maintain the standard elements of the program (ie, educational content, a peer support system, a lifestyle coach) but can overcome the logistical or structural barriers to participation or program delivery that hamper the scalability and access to in-person DPPs (eg, transportation to a physical location, time away from work or private obligations, need for a physical locations suitable to hold group sessions, investment of DPP staff time regardless of participant attendance). Similar to in-person programs, digital DPPs have been tested in workforce populations, and have shown comparable clinical performance to in-person programs.^16^,^17^

The economic value of traditional, group-based, in-person DPPs has been demonstrated in cost-effectiveness and health care cost saving studies, with estimates of savings in the range of US$3000+ within 3 years.^18^–^20^ However, very few cost analyses of workplace-based DPPs have been performed.^18^,^21^ To date, there has been no known rigorous economic analysis of a digital DPP in a worksite population. The field of digital DPP is relatively young, with commercially available digital DPPs only available for widespread use in the past 5 years.^18^ Therefore, the purpose of this study was to examine health care utilization and health care economic outcomes of a large workforce that participated in a digital DPP as a health care benefit from their employer.

## METHODS

This study was an observational analysis of health care claims data among a population of adults with private insurance through their employer. Longitudinal data of annual health care claims were used to examine differences in health care costs and utilization for participants of a digital DPP compared to matched, nonparticipating individuals across the 12 months preceding digital DPP enrollment (the baseline year) and 24 months after program enrollment. Utilizing MarketScan^®^ data, a comparison group of privately insured, working adults who did not participate in the digital DPP was constructed through propensity score matching. The study was approved by the employer Human Studies Review Board with a waiver of consent approved for use of retrospective data that were not originally collected for research purposes.

### Setting

The Dow Company is a global materials science innovations and solutions company headquartered in Midland, MI. Dow operates 113 manufacturing sites in 31 countries, and employs approximately 37 000 people, approximately 17 000 of whom work in the United States. The average United States employee is 44 years old, with an average company tenure of 13 years. The workforce spans several job categories and includes both manufacturing and nonmanufacturing roles.

### Participants

All US-based employees and their spouses/domestic partners (if covered on Dow’s self-insured health plans) were offered the opportunity to participate in the digital DPP if they were determined to be clinically eligible and approved for coverage. Program eligibility requirements included: 18 years of age or older; body mass index (BMI) greater than or equal to 24 kg/m^2^ (or 22 kg/m^2^ if the person endorses Asian racial identity); able to engage in light physical activity; and at risk for T2D as evidenced by (a) a blood-based laboratory test in the prediabetic range (fasting blood glucose 100–125 mg/dL, hemoglobin A1c 5.7–6.4%, or oral glucose tolerance test 140–199 mg/dL), or (b) diagnosis of prediabetes or previous diagnosis of gestational diabetes, or (c) elevated score on a diabetes risk screener.^22^ Participants were excluded based on the following criteria: diagnosis of Type 1 or 2 diabetes prior to enrollment; under treatment for cancer or an acute medical/psychiatric condition that would prohibit full participation; recent cardiac event (such as transient ischemic attack, stroke, acute myocardial infarction, or cardiac surgery), currently pregnant or planning to become pregnant; scheduled for bariatric surgery or recently had bariatric surgery; or other medical conditions that would preclude participation in lifestyle changes and weight reduction.

**Figure 1:**
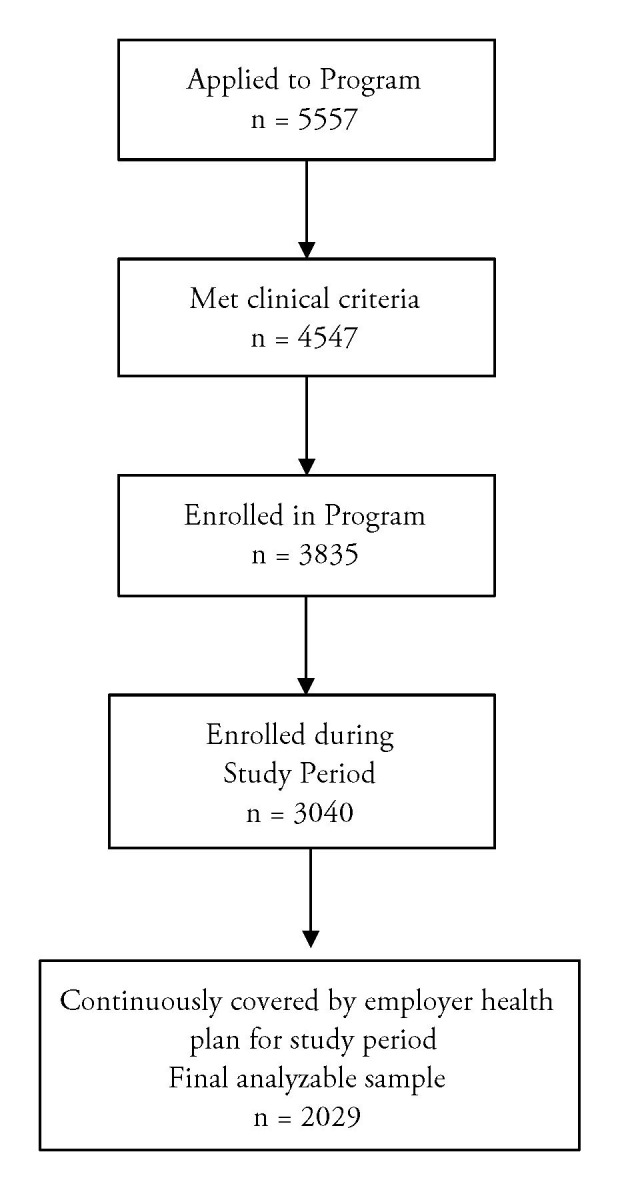
Participant Flowchart

To be included in analyses, participants had to be clinically eligible based on the aforementioned criteria and have continuous employment and health care coverage by the employer for the entire study period. Enrollments began in April of 2015; all eligible participants enrolled through March 2017 were included in the initial pool. Table 1 shows the inclusion and exclusion criteria of the digital DPP participants and their matched controls. A total of 5557 individuals applied to participate in the digital DPP during the eligible time frame. From these applications, 1010 were ineligible either due lack of meeting inclusion criteria or endorsing a medical exclusion criterion. An additional 1101 were removed from analysis because they lacked continuous employment and/or coverage for the study period. Another 795 were removed because they enrolled after March 2017 and did not have 24 months of post-enrollment data at the time of analysis. Finally, 712 started but did not finish the enrollment process and were not included in analyses. A total of 2029 people enrolled and participated in the digital DPP. The participant flow chart is summarized in Figure 1.

Each participant’s calendar start date in the program was used as their study index date. The preceding 12 months before index date composed the baseline year. The program spanned 12 months, comprising the treatment year. Months 13 to 24 comprised the follow-up year. Data were limited to only those participants who contributed health care claims data for the entire study period of baseline through follow-up year.

### Matched Comparison Group

MarketScan^®^ is a comprehensive, proprietary collection of linked health databases of adults in the United States. Data include health care claims data including utilization, medical diagnoses, source of insurance, and actual paid costs of enrollees in commercial, private health insurance plans sponsored by more than 150 large and medium-size employers in the United States. A matched comparison group was constructed from MarketScan for benchmarking purposes. An initial pool of 37 786 people was gathered from MarketScan data. Comparators were chosen based on comparable clinical eligibility, individual demographics, initial health status, baseline health care utilization, and employer characteristics, but comparators were confirmed to have not received the digital DPP benefit or similar program during the study period. Seven employer firms were selected for similar workforce composition and per-enrollee medical and prescription drug spending from all of the employers included in the IBM Watson Health MarketScan Database within the years of 2015 to 2018. Data from digital DPP participants in the baseline year were used for creating the matches. A match ratio of 1:1 was used. In situations with more than one match for a digital DPP participant, we randomly selected one comparison for inclusion in the analytic sample for this study. Only two cases were unable to be matched to MarketScan data and were excluded; 99% of the participants were matched to a comparison subject. The resulting analyses included 2027 digital DPP participants and 2027 matched comparison nonparticipants.

### Digital DPP

The digital DPP is a commercially available product offered by a digital health care company (Omada Health, Inc., San Francisco, CA). The program is recognized by the CDC Diabetes Prevention Recognition Program.^7^ Participants receive support and guidance from a trained lifestyle coach; inclusion in an online peer support forum; digital tools to track weight, physical activity, and eating patterns; and a CDC-approved behavior change curriculum. Participants are assigned to a remote lifestyle coach who communicates through private messages on the online platform. Users are connected to peers through a closed online forum where they could post comments and questions, engage in health coach–moderated discussions, and provide social support to one another. Program participants asynchronously access interactive curriculum lessons through their device of choice (computer, tablet or smartphone). Lessons cover a range of topics from the physiology of diabetes to behavior change topics.

The lifestyle coaches are employed by the company, have a minimum of a bachelor’s degree, and undergo required CDC Lifestyle Coach training from an approved Master Trainer prior to working with participants. Coaches are responsible for monitoring and encouraging lesson completion and health behavior tracking, facilitating discussions on the group forum, and providing individualized, private guidance and support for participants. Throughout the program, participants are asked to track their weight using a cellularly connected digital scale (BodyTrace, Inc., New York, NY), and track their daily meals and physical activity. Users’ weight, activity, and meal tracking data are displayed on each participant’s personal dashboard on the digital platform and shared with their coach. The program is structured along an initial 16-week intensive phase focusing on weight loss, and a subsequent 36-week phase focusing on long-term weight maintenance and sustainment of health behavior changes, for a total of 12 months of programming. Program access remains after 12 months, but no new curriculum lessons are offered; participants retain access to a health coach, the library of archived lessons, an active peer group forum, and the health behavior tracking tools.

### Measures

#### DDP Program Outcomes

To assess the impact of the digital DPP on the biological mechanism of action to prevent progression to T2D (ie, weight loss), we calculated the rate of change in weight from baseline to 16 weeks into the digital DPP program (end of the intensive phase), at 26 weeks (treatment year mid-point), and 52 weeks (end of the full treatment year). Average lesson completion summed across the intensive phase was calculated as a proxy for program engagement.

#### Health care Utilization and Expenditure

Administrative claims data were used to identify the following indicators of health care expenditure: all-cause allowed amount per year for services provided under medical coverage and filled pharmacy prescriptions (medical service and pharmacy analyzed separately and combined); allowed amount for inpatient hospitalization services, allowed amount for outpatient services, and allowed amount for filled pharmacy prescriptions. Amounts represent the summed eligible amounts for the services after applying pricing guidelines, but before deducting third party, copayment, coinsurance, or deductible amounts. In addition, metrics were constructed to capture the total percent of the sample with a medical or pharmacy claim in each year of analysis (analyzed separately and in combination).

The following measures of health care utilization were used: all-cause outpatient visits per year; emergency room (ER) visits per year; inpatient admissions per year and total number of days admitted per year; and days of prescription (Rx) supply filled (ie, number of days of drug therapy covered by a filled prescription). Visits were counted based on a combination of unique patient and service date.

All-cause utilization was examined for each metric as participants were confirmed to not have a pre-existing diabetes diagnosis at study entry. To examine the rate of progression to diabetes, we calculated the percent of each sample with a principal diagnosis of T2D during each year of the study period. We also pooled utilization and cost metrics where the principal diagnosis related to the service was T2D, hypertension (HTN), coronary artery disease (CAD), or congestive heart failure (CHF). We included annual claims from 12 months prior to index date until 24 months after the index date. Program costs for the digital DPP were not included in health care expenditures and were billed directly to the employer.

### Analytic Plan

A pre-post analysis was conducted to examine the percent of initial weight lost by the digital DPP participants from baseline to Week 16, 26, and 52. An average was calculated of the programmatic lessons completed in the first 16 weeks. In the analytic sample of health care claims data for the digital DPP participants and the matched controls, difference-in-differences regression equations were performed on patient-year level data to identify the effects of the digital DPP on the outcome variables. Regression models were used to determine confidence intervals and statistical significance of effects using the following parameters: *Y* = a + *β*1Digital DPP + *β*2Year1 + *β*3*β*1Digital DPP × Year1 + *β*4Year2 + *β*5*β*1Digital DPP × Year2 + *β*6X. *Y* is the outcome for each enrollee; digital DPP is an indicator for whether an individual participated in the digital DPP program; Year1 is an indicator for whether the observation is in the first post-period year and Year2 is an indicator for whether the observation is in the second post-period year; X is a vector of the baseline covariates for age group, male gender, relationship to employer, health plan type, geographic region, rural indicator, comorbidity index, and psychiatric diagnostic group. Effects are measured by the interactions of these dummies with the parameters *β*3 and *β*5. Utilization outcomes were estimated with a negative binomial distribution and a log link. Cost outcomes were estimated with a gamma distribution and a log link. Patient diagnosis incidence outcomes were estimated with a binomial distribution and a logit link. Analyses were performed using WPS analytical software.

## RESULTS

Table 2 shows the comparability of the samples pre- and post-matching. As seen in Table 2, post-matching comparisons reveal that the groups have similar age, sex, race/ethnicity, comorbidity indexes, and health care costs and utilization, which shows that the matching was largely successful, and that the matched controls were reasonable comparators for the participants. The two groups did differ slightly in terms of education levels and race.

### Digital DPP Program Engagement

On average, digital DPP participants completed 10.6 lessons (SD=5.8) out of 16 during the intensive phase of the program. Sixty three percent of the full population completed at least nine lessons.

### Program Outcomes

Among the digital DPP participants, the average weight loss at the end of the intensive phase of the program was 4.3% (SD=5.2%); with 35% of participants achieving at least a 5% weight loss. Weight loss persisted at 4.3% at Week 26, the mid-point of the active treatment year and 2 months into the maintenance phase of the program. This weight loss is similar to that seen in other nationally recognized DPP programs.^23^ The 12-month weight loss outcome was 3.5%.

### Cost Differences

As seen in Table 3, in the year after digital DPP enrollment, the digital DPP group had an all-cause, allowed amount of aggregated medical and pharmacy expenditures that were significantly less than the matched comparison group, with an annual cost difference of −US$1169 per participant (*P* < .05). The savings were primarily from less expenditures for inpatient care (−US$699), with outpatient care (−US$308), and pharmacy (−US$46) contributing to the cost reductions. Pharmacy costs in Year 1 were not significantly different. Average diabetes prevention program cost per participant was US$571, resulting in average net health care expenditure savings of US$598 per participant in Year 1. A trend in reduced expenditures was also seen in Year 2 (−US$630), though not to the same magnitude as the first year and was not statistically significant (*P* = .21). There was a trend of a higher percentage of adults from the matched comparison group with a medical or pharmacy claim in Year 1 relative to the digital DPP group (*P* = .07), with more subjects with a medical claim as the main differentiator (*P* = .04).

### Health care Utilization

Consistent with the patterns in expenditures, there was a significantly reduced amount of health care utilization in inpatient care for the digital DPP group in Year 1. Both the average total number of inpatient admissions per person and the length of stay per person were significantly reduced among the digital DPP participants relative to their matched controls. The digital DPP group also had a smaller total percent of the population with medical and pharmacy claims per year compared to the matched group. The comparison group had more outpatient visits coded to a principal diagnosis of T2D, HTN, CAD, or CHF across both Year 1 (29% more visits compared to digital DPP participants, *P* = .03) and Year 2 (23% more) compared to the digital DPP participants (both *P* values < .05). In Year 2, the comparison group had greater expenditures for pharmacy prescriptions coded to T2D, HTN, CAD, or CHF compared to the DPP group (*P* = .05). The frequency of ER or inpatient admissions coded to these conditions was extremely small for both years.

### Diagnostic Differences

While the progression to T2D diagnosis was small across the study period (less than 3% per year combined across groups), there was a reduced rate of progression in the digital DPP group (−22%) compared to the matched control in Year 1, but the differences were not statistically significant (*P* = .26).

## DISCUSSION

This analysis found a financially meaningful difference in the short-term for digital DPP participation. Digital DPP participants exhibited lesser spend and lower volume of health care use in Year 1 following initiation of the program relative to a rigorously matched comparison sample. The primary impact in health care expense appeared to be from reduced inpatient hospitalizations (both in terms of frequency of admissions and length of stay). More frequent inpatient hospitalizations and longer inpatient stays resulted in greater total spending among the untreated group. Though the magnitude was small, the untreated group also had a greater number of subjects with any kind of medical claim in the first year, more outpatient expenditures/utilization related to T2D or cardiovascular disease relative to the DPP participants in both years and more pharmacy care expenditures/utilization related to T2D or cardiovascular disease relative to the DPP participants in the second year.

While significant savings were evident within the first year, the Year 2 savings did not reach statistical significance, though there was a trend toward the intervention group having fewer expenditures in Year 2. The lack of significance in Year 2 could indicate that the greatest benefit is obtained during the most intensive treatment year, whereas in maintenance years, the magnitude or volume of economic impact is smaller. Longer term follow-up and examination of accumulated economic benefit over several years would help better capture the full impact of the program and durability of savings.

Two years (1 treatment year and 1 year of follow-up) appeared a bit premature to see substantive progression to diabetes in a population of this size. While we did see 22% fewer cases among the DPP group, the combined incidence across the study groups was small during the study timeframe. Most research in the DPP space examines progression to diabetes beyond a 2-year time frame,^24^,^25^ and thus our ambitious time frame may have been too soon to see sizable change in the sample size. Despite that, the initial separation of progression rates between the two groups supports the beneficial impact of the DPP on risk reduction in the short-term.

We could not identify economic analyses of diabetes prevention programs that focus on digital delivery, worksite populations, or derive data from actual health insurance claims; therefore, there are limited data to compare these findings relative to the field.^19^ However, HELP-PD was an in-person, group-based, translational DPP program that examined costs over a 2-year time frame, and found an accumulated 2-year savings of US$2277 in direct medical care among the intervention group relative to the usual care group.^26^ One economic simulation predicted an accumulated average of US$3070 in gross direct and indirect savings over 2 years, with nonmedical benefit accounting for the majority of savings.^27^ Another simulation did not predict savings within the first 10 years.^28^ The results from the current analysis appear within the range of savings found from in-person programs, but more cost-savings economic analyses of both digital and in-person, translational DPPs are clearly needed.

### Limitations

The study was an observational study, and thus the participants self-selected into participating in the program. This potential selection bias may have contributed to the positive health gains seen in the treated population. However, this was a real-world case study of a preventive health service implemented under standard delivery conditions. Qualified, eligible members are typically targeted to participate without restriction in services that may benefit them. Additionally, the rigorous matching process accounted for a multitude of personal, socioeconomic, and environmental variables, and the high precision in matching is reassurance that the study cohorts had minimal differences at the outset.

Another limitation was that we observed participants for only 24 months. This is a short and ambitious time frame to find significant variation in health status and progression to a chronic condition among a population that was generally healthy and only at risk for chronic conditions and may account for the small rate of incidence in chronic disease. Additionally, our methodology of classifying utilization based on principle diagnostic code associated with the visit may have resulted in an under-estimate of incident cases of chronic disease; diabetes may have been detected in visits, but if it was not the initiating factor for the visit, it would have gone undetected in our analysis. Longer term follow-up of study participants and inclusion of subsequent cohorts would be useful for tracking the rate of diabetes progression. Longer follow-up would also be instructive for evaluating the impact of diabetes prevention on longer-term cost outcomes, as economic simulation models suggest that greater savings may be expected up to 10 years out from a treatment phase.^27^,^29^

Another limitation worth noting is that our analysis plan called for estimating adjusted outcomes using generalized linear models. Specifically, cost outcomes were estimated with a gamma distribution and a log link. The impact estimate generated from that model (ie, exponentiated coefficients) is not linear or additive in nature. Instead, the estimate is interpreted on a multiplicative scale and statistical testing was applied to that scale. To further test the statistical significance of the cost results, researchers may wish to apply analyses that test the marginal effects of the intervention, such as differences in adjusted costs between treatment and comparison groups, using bootstrapping methods.

Economic analyses are often explored after clinically meaningful findings have been seen; as a result, these investigations are not intentionally planned with sufficient statistical power.^30^ The same principle holds true in this current analysis, as it was based on naturally occurring program enrollments and the total volume of participants was not prospectively determined with specific statistical power considerations in mind. However, it is fair to argue that statistical significance of an inferential test can be scientifically important, but not necessarily valuable for pragmatic decisions. The overall net benefit is an important decision-making factor that deserves consideration.

## CONCLUSION

Our results demonstrate that net positive economic benefits can been seen from digital DPP within a 1-year time horizon. With broadened coverage and uptake by employers and health plans, the population health benefit of digital DPPs will continue to grow. Given the scalability and reduction in access barriers, including digital methods to expand reach and access of preventive services makes practical sense, and the results of this study suggest it will also make economic sense.

## Figures and Tables

**Table 1:** Inclusion & Exclusion Criteria

Inclusion Criteria	
Continuously enrolled in an active, self-insured plan for 36- month study time frame (April 2015 to March 2018)	
Age 18 to 64 during the study time frame	
Met one of the following: Study group: Participated in the digital DPP program with enrollment dates between April 2016 and March 2017 Control group: Met the clinical criteria for program eligibility based on biometric, self-reported health risk assessment, or claims data (BMI ≥ 25 and one of the following: prediabetes, hypertension, dyslipidemia, or tobacco use) in baseline year	
**Exclusion Criteria**	
Did not meet inclusion criteria for the study	
Type 2 diabetes, Type 1 diabetes, or diabetic drug prescription during the baseline year	
TIA/stroke, AMI, CHF hospitalization, cardiac surgery, or bariatric surgery during the baseline year	
Pregnancy, eating disorder, alcohol/substance abuse, organ transplant, cancer treatment, dialysis treatment or Type 1 diabetes during program year 1	

Abbreviations: AMI, acute myocardial infarction; CHF, congestive heart failure; DPP, Diabetes Prevention Program; TIA, transient ischemic attack.

**Table 2:** Baseline Year Matching Characteristics, Pre- and Post-Match

Baseline	Digital DPP Pre-Match	MarketScan Pre-Match	Digital DPP Post-Match	MarketScan Post-Match	*P* value
**Members**	2029	37 786	2027	2207	

**Age Group**					0.96
18–34	18%	18%	17.9%	17.5%	
35–44	24%	28%	24.1%	24.3%	
45–54	43%	34%	43.2%	43.8%	
55–65	15%	21%	14.8%	14.5%	

**Geographic Region**					0.65
North East	7%	9%	7.5%	7.0%	
North Central	48%	50%	47.6%	46.2%	
South	44%	39%	44.4%	46.3%	
West	1%	2%	0.5%	0.5%	

**Gender**					0.87
Female	52%	26%	51.5%	51.8%	
Male	48%	74%	48.5%	48.2%	

**Relationship**					0.69
Employee	88%	96%	87.6%	88.4%	
Spouse	12%	4%			
Dependent	1%	0%			

**Plan Type**					0.68
HMO/EPO	23%	13%	77.5%	78%	
PPO/POS	77%	87%			

**Rural Area Indicator**					0.60
No	94%	82%	95.6%	94%	
Yes	6%	18%	6.4%	6.0%	

**Median Household Income**^a^					0.10
<US$46 500	34%	12%	34%	10%	
US$46 500–US$56 999	16%	36%	16%	35%	
US$57 000–US$68 999	31%	37%	31%	43%	
> US$69 000	19%	15%	19%	12%	

**Percent No High School**	4.7%	3.9%	4.6%	3.7%	*<.01

**Percent White**	79%	78%	79%	77%	*<.01

**Allowed Amount Med & Rx**					.89
US$0	7%	10%	6.8%	6.0%	
US$1–US$5000	66%	68%	66.5%	66.8%	
US$5001–US$25 000	23%	18%	22.6%	23.0%	
US$25 000–US$50 000	3%	2%	2.6%	2.8%	
>US$50 000	1%	1%	1.5%	1.4%	

**Charlson Cormorbidity Index**					.35
0 risks	88%	88%	88.0%	87.3%	
1–2 risks	11%	11%	11.1%	12.1%	
3+ risks	1%	1%	0.9%	0.6%	

**# Psychiatric Diagnostic Groups**					.84
0	79%	83%	79.1%	78.5%	
1	14%	13%	14.2%	14.3%	
2+	7%	5%	6.8%	7.2%	

**Clinical Indicators**					
CAD Diagnosis	1.3%	2.8%	1.3%	1.3%	.99
CHF Diagnosis	0.1%	0.3%	0.1%	0.1%	.65
HTN Diagnosis	15%	26%	14.9%	15.9%	.38

Abbreviations: CAD, coronary artery disease; DPP, Diabetes Prevention Program; EPO, exclusive provider organization; HMO, health maintenance organization; HTN, hypertension; POS, point of service; PPO, preferred provider organization.

a Income based on census data for 3-digit zip code

**Table 3:** Differences-in-Differences Outcomes of Health care Costs and Utilization Between DPP Participants (n = 2027) and Matched Comparisons (n = 2027)

	Year 1 (Treatment Year)					Year 2				
	Adjusted Mean Diff	Interaction Coefficient	Lower 95% CI	Upper 95% CI	*P* Value	Adjusted Mean Diff	Interaction Coefficient	Lower 95% CI	Upper 95% CI	*P* value
Allowed Amount Med + Rx	−US$1169	−0.23	−0.41	−0.05	0.01	−US$630	−0.12	−0.30	0.07	0.21
Inpatient Allowed Amount	−US$699	−1.39	−2.24	−0.55	0.001	−US$168	−0.20	−0.90	0.50	0.58
Outpatient Allowed Amount	−US$308	−0.09	−0.26	0.08	0.29	−US$275	−0.08	−0.26	0.09	0.36
Rx Allowed Amount	−US$46	−0.04	−0.40	0.32	0.83	−US$210	−0.19	−0.61	0.23	0.38
Percent of Members with a Medical or Rx Claim	−2%	−0.27	−0.57	0.02	0.07	−1%	−0.17	−0.47	0.14	0.28
Percent of Members with a Medical Claim	−2%	−0.27	−0.54	−0.01	0.04	−1%	−0.19	−0.46	0.09	0.18
Percent of Members with an Rx Claim	1%	0.05	−0.13	0.22	0.58	3%	0.13	−0.05	0.31	0.15
Admissions (#)	−0.02	−1.01	−1.57	−0.44	0.0005	−0.01	−0.32	−0.84	0.20	0.23
Days of Admissions (#)	−0.06	−1.47	−2.23	−0.71	0.0002	−0.02	−0.32	−1.15	0.50	0.44
ER Visits (#)	0.01	0.07	−0.17	0.30	0.59	0.00	−0.01	−0.26	0.24	0.96
Outpatient visits (#)	−0.25	−0.02	−0.09	0.05	0.50	0.58	0.05	−0.02	0.13	0.16
All-Cause Rx Supply	−3.24	−0.01	−0.05	0.03	0.69	−11.31	−0.03	−0.08	0.02	0.29
Type 2 Diabetes, Cardiac, and Hypertension Admits	0.00	−57	−1191	1076	0.92	0.00	0.00	−3.39	3.39	1.00
Type 2 Diabetes, Cardiac, and Hypertension ER Visits	0.00	−0.18	−2.15	1.78	0.86	0.00	0.12	−2.49	2.72	0.93
Type 2 Diabetes, Cardiac, and Hypertension Outpatient Visits	−0.09	−0.34	−0.56	−0.12	0.03	−0.07	−0.26	−0.48	−0.04	0.02
Type 2 Diabetes, Cardiac, and Hypertension Rx Days Supply	−5.11	−0.04	−0.12	0.04	0.31	−12.74	−0.11	−0.21	0.00	0.05
Percent with Type 2 Diabetes Diagnosis	−22%	−0.25	−0.69	0.19	0.26	0%	0.00	0.00	0.00	1.00
